# Supplementary material for: Modeling glioblastoma heterogeneity as a dynamic network of cell states
Source: Mol Syst Biol. 2021 Sep 16;17(9):e10105. doi: 10.15252/msb.202010105 (PMC8444284; doi:10.15252/msb.202010105)
Supplement: Supplementary file 6 — Source Data for Figure 5 [file MSB-17-e10105-s004.zip › Figure5A_sourcedata/GSEA_3017/nosigdb_stateB.GseaPreranked.1622795021725/RICHARDS_NOWAKOWSKI_SCIENCE_2017_RG_EARLY_UPREG.html]

Details for gene set RICHARDS\_NOWAKOWSKI\_SCIENCE\_2017\_RG\_EARLY\_UPREG[GSEA]

|  || Dataset | state43017 |
| Phenotype | NoPhenotypeAvailable |
| Upregulated in class | na\_pos |
| GeneSet | RICHARDS\_NOWAKOWSKI\_SCIENCE\_2017\_RG\_EARLY\_UPREG |
| Enrichment Score (ES) | 0.51755714 |
| Normalized Enrichment Score (NES) | 3.1156693 |
| Nominal p-value | 0.0 |
| FDR q-value | 0.0 |
| FWER p-Value | 0.0 |
Table: GSEA Results Summary

  

Fig 1: Enrichment plot: RICHARDS\_NOWAKOWSKI\_SCIENCE\_2017\_RG\_EARLY\_UPREG      
 Profile of the Running ES Score & Positions of GeneSet Members on the Rank Ordered List

  

| PROBE | GENE SYMBOL | GENE\_TITLE | RANK IN GENE LIST | RANK METRIC SCORE | RUNNING ES | CORE ENRICHMENT || 1 | HIST1H4C |  |  | 0 | 1.373 | 0.0214 | Yes |
| 2 | HIST1H1B |  |  | 1 | 1.272 | 0.0411 | Yes |
| 3 | HIST1H1D |  |  | 2 | 1.180 | 0.0595 | Yes |
| 4 | TOP2A |  |  | 3 | 1.079 | 0.0763 | Yes |
| 5 | HIST1H1C |  |  | 4 | 1.041 | 0.0924 | Yes |
| 6 | HIST1H1E |  |  | 5 | 0.983 | 0.1077 | Yes |
| 7 | RRM2 |  |  | 7 | 0.875 | 0.1197 | Yes |
| 8 | UBE2C |  |  | 11 | 0.802 | 0.1275 | Yes |
| 9 | TPX2 |  |  | 12 | 0.801 | 0.1399 | Yes |
| 10 | NUSAP1 |  |  | 13 | 0.801 | 0.1524 | Yes |
| 11 | HMGB2 |  |  | 14 | 0.786 | 0.1646 | Yes |
| 12 | NCAPG |  |  | 16 | 0.778 | 0.1751 | Yes |
| 13 | SPC25 |  |  | 17 | 0.763 | 0.1870 | Yes |
| 14 | PRC1 |  |  | 18 | 0.756 | 0.1987 | Yes |
| 15 | KIF23 |  |  | 24 | 0.721 | 0.2020 | Yes |
| 16 | ASPM |  |  | 26 | 0.695 | 0.2113 | Yes |
| 17 | NDC80 |  |  | 27 | 0.695 | 0.2221 | Yes |
| 18 | ATAD2 |  |  | 28 | 0.685 | 0.2327 | Yes |
| 19 | KIF15 |  |  | 29 | 0.684 | 0.2433 | Yes |
| 20 | ARHGAP11A |  |  | 30 | 0.683 | 0.2540 | Yes |
| 21 | CENPF |  |  | 34 | 0.670 | 0.2596 | Yes |
| 22 | KIF4A |  |  | 37 | 0.662 | 0.2668 | Yes |
| 23 | ANLN |  |  | 39 | 0.659 | 0.2754 | Yes |
| 24 | RAD51AP1 |  |  | 40 | 0.657 | 0.2857 | Yes |
| 25 | SMC4 |  |  | 41 | 0.655 | 0.2958 | Yes |
| 26 | CENPE |  |  | 43 | 0.654 | 0.3044 | Yes |
| 27 | PRR11 |  |  | 45 | 0.649 | 0.3129 | Yes |
| 28 | DLGAP5 |  |  | 46 | 0.647 | 0.3230 | Yes |
| 29 | KIF18A |  |  | 48 | 0.639 | 0.3314 | Yes |
| 30 | NCAPG2 |  |  | 50 | 0.636 | 0.3397 | Yes |
| 31 | CKAP2 |  |  | 53 | 0.628 | 0.3463 | Yes |
| 32 | CCNA2 |  |  | 54 | 0.626 | 0.3560 | Yes |
| 33 | CKAP2L |  |  | 59 | 0.611 | 0.3592 | Yes |
| 34 | CENPU |  |  | 60 | 0.605 | 0.3686 | Yes |
| 35 | CENPK |  |  | 62 | 0.603 | 0.3764 | Yes |
| 36 | TYMS |  |  | 63 | 0.601 | 0.3857 | Yes |
| 37 | BUB1B |  |  | 64 | 0.596 | 0.3950 | Yes |
| 38 | KIF2C |  |  | 66 | 0.591 | 0.4026 | Yes |
| 39 | SMC2 |  |  | 68 | 0.586 | 0.4101 | Yes |
| 40 | CDK1 |  |  | 69 | 0.585 | 0.4192 | Yes |
| 41 | CKS1B |  |  | 72 | 0.575 | 0.4250 | Yes |
| 42 | RACGAP1 |  |  | 75 | 0.572 | 0.4307 | Yes |
| 43 | MAD2L1 |  |  | 77 | 0.569 | 0.4380 | Yes |
| 44 | DBF4 |  |  | 79 | 0.567 | 0.4452 | Yes |
| 45 | CKS2 |  |  | 83 | 0.558 | 0.4491 | Yes |
| 46 | ECT2 |  |  | 84 | 0.558 | 0.4578 | Yes |
| 47 | KIF14 |  |  | 85 | 0.557 | 0.4665 | Yes |
| 48 | SPC24 |  |  | 91 | 0.544 | 0.4670 | Yes |
| 49 | CCNB1 |  |  | 93 | 0.544 | 0.4739 | Yes |
| 50 | PBK |  |  | 95 | 0.540 | 0.4807 | Yes |
| 51 | CDKN3 |  |  | 108 | 0.513 | 0.4697 | Yes |
| 52 | CEP135 |  |  | 109 | 0.513 | 0.4777 | Yes |
| 53 | UBE2T |  |  | 110 | 0.512 | 0.4856 | Yes |
| 54 | CDCA3 |  |  | 111 | 0.511 | 0.4935 | Yes |
| 55 | BIRC5 |  |  | 116 | 0.504 | 0.4951 | Yes |
| 56 | CEP152 |  |  | 119 | 0.502 | 0.4997 | Yes |
| 57 | DUT |  |  | 124 | 0.494 | 0.5010 | Yes |
| 58 | ITGB3BP |  |  | 128 | 0.490 | 0.5039 | Yes |
| 59 | GMNN |  |  | 135 | 0.484 | 0.5019 | Yes |
| 60 | TTK |  |  | 137 | 0.482 | 0.5079 | Yes |
| 61 | FANCD2 |  |  | 149 | 0.461 | 0.4976 | Yes |
| 62 | KIF22 |  |  | 152 | 0.459 | 0.5016 | Yes |
| 63 | KNSTRN |  |  | 155 | 0.456 | 0.5055 | Yes |
| 64 | SPAG5 |  |  | 156 | 0.455 | 0.5126 | Yes |
| 65 | BRCA1 |  |  | 171 | 0.440 | 0.4973 | Yes |
| 66 | RBBP8 |  |  | 176 | 0.436 | 0.4977 | Yes |
| 67 | MKI67 |  |  | 179 | 0.436 | 0.5013 | Yes |
| 68 | USP1 |  |  | 185 | 0.430 | 0.5001 | Yes |
| 69 | MIS18BP1 |  |  | 186 | 0.430 | 0.5068 | Yes |
| 70 | BARD1 |  |  | 189 | 0.427 | 0.5103 | Yes |
| 71 | MND1 |  |  | 193 | 0.424 | 0.5121 | Yes |
| 72 | MCM4 |  |  | 199 | 0.418 | 0.5107 | Yes |
| 73 | SYNE2 |  |  | 203 | 0.413 | 0.5124 | Yes |
| 74 | ZWINT |  |  | 212 | 0.407 | 0.5060 | Yes |
| 75 | H2AFZ |  |  | 216 | 0.405 | 0.5076 | Yes |
| 76 | GPC4 |  |  | 217 | 0.404 | 0.5139 | Yes |
| 77 | TPM1 |  |  | 225 | 0.397 | 0.5090 | Yes |
| 78 | TIMELESS |  |  | 228 | 0.395 | 0.5119 | Yes |
| 79 | EZH2 |  |  | 236 | 0.388 | 0.5069 | Yes |
| 80 | PCNA |  |  | 242 | 0.386 | 0.5050 | Yes |
| 81 | HMGN2 |  |  | 243 | 0.386 | 0.5110 | Yes |
| 82 | RTKN2 |  |  | 248 | 0.379 | 0.5106 | Yes |
| 83 | CCNB2 |  |  | 250 | 0.379 | 0.5149 | Yes |
| 84 | HEY1 |  |  | 253 | 0.376 | 0.5176 | Yes |
| 85 | CENPW |  |  | 267 | 0.366 | 0.5027 | No |
| 86 | ZFP36L2 |  |  | 279 | 0.358 | 0.4908 | No |
| 87 | ANP32E |  |  | 280 | 0.358 | 0.4964 | No |
| 88 | PSIP1 |  |  | 284 | 0.357 | 0.4972 | No |
| 89 | HAUS8 |  |  | 293 | 0.353 | 0.4900 | No |
| 90 | CDC25C |  |  | 307 | 0.343 | 0.4748 | No |
| 91 | CENPN |  |  | 309 | 0.343 | 0.4786 | No |
| 92 | ABHD3 |  |  | 310 | 0.343 | 0.4839 | No |
| 93 | DEK |  |  | 316 | 0.341 | 0.4813 | No |
| 94 | PRDX6 |  |  | 323 | 0.338 | 0.4770 | No |
| 95 | BTG3 |  |  | 337 | 0.331 | 0.4616 | No |
| 96 | XPO1 |  |  | 365 | 0.321 | 0.4239 | No |
| 97 | RHEB |  |  | 404 | 0.307 | 0.3685 | No |
| 98 | PTTG1 |  |  | 413 | 0.302 | 0.3605 | No |
| 99 | AHI1 |  |  | 425 | 0.299 | 0.3478 | No |
| 100 | IQGAP3 |  |  | 447 | 0.294 | 0.3191 | No |
| 101 | DYRK1A |  |  | 450 | 0.293 | 0.3205 | No |
| 102 | HSPA4L |  |  | 461 | 0.290 | 0.3092 | No |
| 103 | NUP155 |  |  | 468 | 0.288 | 0.3042 | No |
| 104 | TTYH1 |  |  | 469 | 0.287 | 0.3086 | No |
| 105 | NUP50 |  |  | 470 | 0.287 | 0.3131 | No |
| 106 | CHEK1 |  |  | 477 | 0.286 | 0.3081 | No |
| 107 | YBX3 |  |  | 519 | 0.278 | 0.2475 | No |
| 108 | CNN3 |  |  | 527 | 0.276 | 0.2407 | No |
| 109 | YAP1 |  |  | 560 | 0.270 | 0.1943 | No |
| 110 | CTNNAL1 |  |  | 570 | 0.269 | 0.1842 | No |
| 111 | CCDC18 |  |  | 586 | 0.267 | 0.1647 | No |
| 112 | PAX6 |  |  | 617 | 0.261 | 0.1212 | No |
| 113 | HMGA2 |  |  | 619 | 0.260 | 0.1237 | No |
| 114 | UHRF1 |  |  | 652 | 0.255 | 0.0770 | No |
| 115 | RPL10A |  |  | 684 | -0.260 | 0.0320 | No |
| 116 | RPL13A |  |  | 685 | -0.260 | 0.0361 | No |
| 117 | RPS25 |  |  | 687 | -0.263 | 0.0386 | No |
| 118 | VIM |  |  | 692 | -0.267 | 0.0364 | No |
| 119 | RPL37 |  |  | 696 | -0.274 | 0.0359 | No |
| 120 | HSP90AB1 |  |  | 701 | -0.277 | 0.0339 | No |
| 121 | RPL5 |  |  | 702 | -0.279 | 0.0382 | No |
| 122 | RPL23 |  |  | 704 | -0.283 | 0.0410 | No |
| 123 | ID4 |  |  | 708 | -0.285 | 0.0407 | No |
| 124 | ZEB1 |  |  | 717 | -0.317 | 0.0330 | No |
| 125 | PNRC1 |  |  | 726 | -0.348 | 0.0257 | No |
| 126 | HSPB1 |  |  | 730 | -0.378 | 0.0269 | No |
| 127 | PPA1 |  |  | 742 | -0.424 | 0.0161 | No |
| 128 | RPS24 |  |  | 757 | -0.595 | 0.0032 | No |
Table: GSEA details [plain text format]

  

Fig 2: RICHARDS\_NOWAKOWSKI\_SCIENCE\_2017\_RG\_EARLY\_UPREG: Random ES distribution      
 Gene set null distribution of ES for **RICHARDS\_NOWAKOWSKI\_SCIENCE\_2017\_RG\_EARLY\_UPREG**
